# A detection and quantification label-free tool to speed up downstream processing of model mucins

**DOI:** 10.1371/journal.pone.0190974

**Published:** 2018-01-09

**Authors:** Sofia B. Carvalho, Ana Sofia Moreira, Joana Gomes, Manuel J. T. Carrondo, David J. Thornton, Paula M. Alves, Julia Costa, Cristina Peixoto

**Affiliations:** 1 iBET, Instituto de Biologia Experimental e Tecnológica, Oeiras, Portugal; 2 Instituto de Tecnologia Química e Biológica António Xavier, Universidade Nova de Lisboa, Oeiras, Portugal; 3 Departamento de Química, Faculdade de Ciências e Tecnologia, Universidade Nova de Lisboa, Monte da Caparica, Portugal; 4 Faculty of Biology, Medicine and Health, University of Manchester, Manchester, United Kingdom; Consiglio Nazionale delle Ricerche, ITALY

## Abstract

Mucins are high-molecular weight glycoproteins (0.25–20 MDa) containing one or more domains that are heavily O-glycosylated. Their implications as targets for cancer treatment have increased the interest in these glycoproteins, mainly in the fields of vaccines and antibodies. However, mucins present high heterogeneity, posing challenges that affect purification processes and quality control analysis. In that sense, it is necessary to develop and improve downstream processes and analytical methods to characterize these products.

Here a tool based on biolayer interferometry analysis to improve mucin’s detection and quantification in a fast, simple and label free-way is presented. Taking advantage of lectin recognition of mucins’ carbohydrate structures, several lectins were evaluated and immobilized on streptavidin biosensors. Different assay conditions were optimized and the most suitable lectin, Aleuria aurantia lectin (AAL), was selected. Bovine Submaxillary Gland and human MUC5B mucins were used as proof of concept and were successfully detected and quantified at different stages of purification. High sensitivity levels were achieved with LOD and LOQ of 3.8 μg mL^-1^ and 11.7 μg mL^-1^ for BSM, and 0.2 μg mL^-1^ and 0.6 μg mL^-1^ for MUC5B. AAL binding specificity was also confirmed with fucose competition assays. Our method represents an advance on mucins detection and quantification since the existing methods present several disadvantages for process development. Hereafter, it can be applied to the optimization of new or already established downstream processes for mucins’ purification.

## Introduction

Mucins are a heterogeneous family of complex high-molecular-weight glycoproteins (0.25–50 MDa), produced by epithelial cells [1]. Being part of the mucosal barrier, they have been associated with several important functions like microenvironment regulation, homeostasis maintenance and cell protection. These glycoproteins have a highly heterogeneous structure, mainly due to a huge variety and extension of O-glycosylation at PTS (proline, threonine and serine) domains via N-acetyl-D-galactosamine (GalNAc) linkage sugar [2]. During the past few decades, several studies have supported mucins clinical significance [3]. This glycoprotein family has been linked to a variety of inflammatory diseases and cancer, making it a good candidate to generate potential cancer vaccines and therapies [4]. However, mucins saccharides heterogeneity poses challenges in the downstream process development and analytical methods [5]. The detection methods available are not suitable for process development because the results are not immediate [6]. The existence of an analytical method that could easily detect and quantify mucins, would have a huge impact for example in the diagnostic of respiratory diseases and in the downstream processes monitoring. As an example, in the literature some results have indicated that one of MUC5B variants predominate in mucus associated with chronic obstructive pulmonary disease (COPD) and cystic fibrosis (CF) [7–9]. Thus, there is a need for a more robust, fast and scalable technique to address the hurdles associated with mucins detection and quantification for diagnosis or as product for the downstream process [10].

Nowadays, biopharmaceutical industry and regulatory agencies constraints require new and improved bioprocesses that must be validated with better analytical tools. These analytics should be time-saving, robust and capable of analysing in-process samples, from crude bulk to purified samples. In the case of mucins bioprocesses, their heterogeneity, complexity and the lack of knowledge on their structure have been imposing numerous challenges that need to be overcome, mainly in their production, characterization and quantification. Several techniques are being applied for mucin’s detection and quantitation using two different approaches: chemical stains, that take advantage of their glycosylated structure; or antibodies specific for known peptide sequences. For chemical detection, the most commonly used methods are high iron diamine (HID), alcian blue (AB) and Periodic Acid/Schiff’s (PAS) reagent. These assays can detect mucins at intermediate purification steps. However, they lack specificity since they stain all glycoproteins [11]. Detection using antibodies is an attractive approach to overcome the specificity problem. However, this strategy is not effective for mucin detection in secretions and cell culture media because antibodies raised against PTS-rich domains can also detect mucins precursors besides the mature mucin.

Here we report a Biolayer interferometry (BLI) analysis assay that uses the Octet^®^ System platform (Fortébio, Inc. Menlo Park, CA) for mucin detection and quantification. This technology was already reported as a successful tool for different complex biomolecules and applications [12–14]. Two distinct mucins were evaluated as models: MUC5B human Mucin and Bovine Submaxillary gland Mucin (BSM). MUC5B, one of the major constituents of the human respiratory tract and one of the responsibles for protecting cell surface from infection, was used as a human model [15]. BSM is produced in the submaxillary glands and it is secreted in the saliva [16]. Since it is commercially available, was chosen for assay design and optimization. MUC5B is heavily O-glycosylated with a high heterogeneity of structures: fucose was found in blood-group H, Lewis^a^, Lewis^b^, Lewis^x^, Lewis^y^ structures, N-acetylneuraminic acid (NeuAc) was mainly α2,3-linked to Gal or α2,6-linked to GalNAcol, the major internal structures were core-type 1 or 2 [17]. In addition, MUC5B is N-glycosylated [18] and C-mannosylated [19]. BSM is also extensively O-glycosylated with blood-group H structures, core-type 1, 2, 3 or 4 internal structures and NeuAc or N-glycolylneuraminic acid (NeuGc) α2,6-linked to GalNAcol [20].

The method described in this work takes advantage of lectin-mucin binding. Lectins are sugar-binding proteins that can recognize even slight variations in carbohydrate structures and be isolated from several origins [21]. Consequently, they have been used for characterization, purification and drug targeting of different glycoprotein classes [22]. Biotinylated lectins can be attached to the surface of a streptavidin functionalized biosensor and then mucins can be associated to these lectins, producing a binding signal.

The use of biosensors to detect glycosylated structures from mucins is an emerging area with different applications. However, only a few detectors were developed and are available for mucins. For example, a label-free electrochemical impedance spectroscopy biosensor with the lectin *Sambucus nigra* agglutinin type I detected the cancer-associated sialyl-Tn antigen in glycoproteins, including up to 40 ng bovine submaxillary mucin [23]. On the other hand, BLI technology allows detection and quantification of mucin sugars in a fast, high throughput, simple and label-free way [24–26]. Moreover, this methodology provides a simple and fast analysis with real-time results when compared to the traditional techniques, for example, an ELISA assay [27]. Additionally, Limit of Detection (LOD) and Limit of Quantitation (LOQ) [28] are in the same order of magnitude of ELISA [29] (nanograms). LOD and LOQ for BSM are 3.54 μg mL^-1^ and 10.72 μg mL^-1^, respectively, and 0.2 μg mL^-1^ and 0.6 μg mL^-1^ for MUC5B. Overall, this strategy is a powerful tool for process monitoring and optimization and can be further applied to mucins’ bioprocess development of new or already established purification processes.

## Materials and methods

### Mucins and lectins samples

Biotinylated lectins evaluated were *Aleuria aurantia* lectin (AAL, 150091), Peanut Agglutinin (PNA, 150061), Wheat Germ Agglutinin (WGA, 150031), *Maackia amurensis* lectin (MAL, 150131), *Sambucus nigra* agglutinin (SNA, 150121), Snowdrop lectin (GNA, 150021). All of them were purchased from Galab Technologies, Germany. Two distinct mucins were used: Bovine Submaxillary Mucin (BSM) (84195-52-8, Sigma Aldrich^®^) and Human 5B Mucin (isolated from human saliva). Saliva was donated by healthy participants who provided their written consent and MUC5B was purified as described previously (Davies et al., 2014 PLOS ONE 9:e108372). Ethical approval for this research was acquired from the University of Manchester Research Ethics Committee (08293).

### Biolayer interferometry

**Fig 1:**
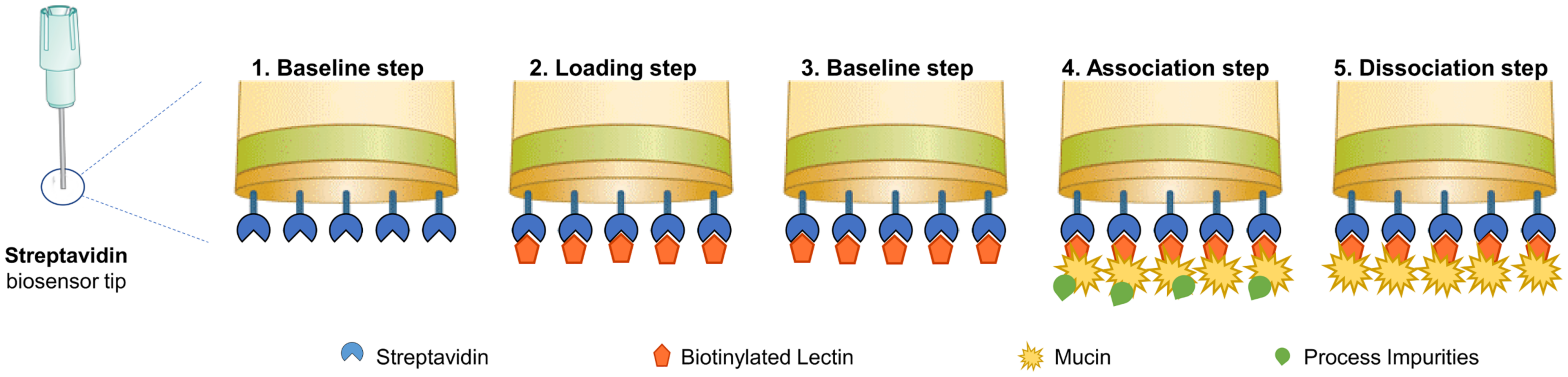
Schematic representation of mucin method. Schematic representation of octet method for detection and quantification of mucins using functionalized Streptavidin biosensors. After an initial baseline step (1) in sample diluent, biotinylated lectins are loaded (2). A second baseline step (3) using sample buffer is performed followed by mucins association (4) to lectins. The final step comprises dissociation of other process components (5).

Mucin detection and quantification were performed using an Octet RED96 System (fortéBIO, Pall Corp., USA). Briefly, biotinylated lectins were diluted with Sample diluent (18–5028, fortéBIO, Pall Corp., USA) and loaded onto High Precision Streptavidin (SAX) Biosensors (18–0037, fortéBIO, Pall Corp., USA), previously hydrated and blocked with the same buffer. Then, a second baseline step was performed to wash unbound lectins. Mucin samples were then associated with the different lectins and association and dissociation profiles were measured (Fig 1). Experiments were performed using the kinetics mode, at 25°C and sample plates were agitated at 1000 rpm. Data Analysis v9.0 software (fortéBIO, Pall Corp., USA) was used for data fitting and mucins’ concentration calculation. Quantifications were performed taking into consideration the initial values (0 to 100 s) of the binding responses. Response value was given by the slope of the line within the window of interest described above. Local partial fitting was applied to association step assuming that dissociation does not reach the pre-association baseline. The mathematical model used assumes a simple 1:1 stoichiometry, fitting only one analyte in solution binding to one binding site on the surface. A Savitzky-Golay filter was applied to smooth the data.

### Statistical analysis

To establish Limit of Detection (LOD) and Limit of Quantitation (LOQ) parameters, the FDA Guidelines were followed (FDA, ICH Guidance for Industry, Q2B Validation of Analytical Procedures: Methodology, 1996). From the several possible approaches, the determination of both limits was defined based on the standard response deviation and the slope of the calibration curve:

$$LOD\;=\;\frac{3.3\sigma }{S}$$ (1)

$$LOQ\;=\;\frac{10\sigma }{S}$$ (2)

where σ is the standard deviation of the response and S the slope of the calibration curve [30]. The standard deviations and coefficients of variation of LOD and LOQ for BSM, used as a model, were calculated using three calibration curves as independent replicates. The errors indicated correspond to two times the standard deviation. For all the calibration curves, the standard error of the estimation associated with the linear regression was calculated (the corresponding error in concentration is indicated). MUC5B was extremely limited as it is a highly purified human sample. Therefore, it was used as proof of concept and one replicate of the calibration curve was done.

### Size exclusion chromatography (SEC)

A BSM solution (4.25 mg mL^-1^) was prepared with 20 mM PIPES, 300 mM NaCl buffer (working buffer), at pH 5, and filtered with a 0.45 μm Minisart^®^ High Flow Hydrophobic PES syringe filter. SEC was conducted using a Superdex 200 Increase 10/300 GL column (GE Healthcare, USA) coupled to an ÄKTA^™^ avant 150 liquid chromatography system (GE Healthcare, USA) equipped with UV and conductivity/pH monitors. System operation and data gathering and analysis were performed using the UNICORN^™^ 6.3 software (GE Healthcare, USA). The column was loaded with 1 mL of BSM at a constant flow rate of 0.6 mL.min^-1^. Working buffer was used as eluent and the eluted fractions were collected for further analysis. Elution of BSM was monitored at 230, 260 and 280 nm.

### SDS-PAGE and lectin blotting

BSM samples were analysed by SDS-PAGE, in 10% acrylamide gels with running buffer 1x (25 mM Tris, 201-064-4 Carl Roth^®^, 192 mM Glycine, 200-272-2 Sigma Aldrich^®^, 0.1% SDS). Samples (15 μL) were loaded and the electrophoresis was run at 180 V for 60 minutes. BSM concentration was 0.17 μg μL^-1^, corresponding to 2.5 μg of mucin loaded. MUC5B concentration was 0.033 μg μL^-1^, corresponding to 0.5 μg of mucin loaded. Proteins from the SDS-PAGE gel were transferred onto polyvinylidene fluoride (PVDF) membranes. Lectin blotting was performed with the lectins described in Biolayer Interferometry section essentially as described before [31] Blots were blocked with 3% Bovine Serum Albumin biotin free (292-322-5 Carl Roth^®^) in Tris Buffered Saline with 0.1% Tween 20 (TBST) for 1 hour, and incubated with each lectin for 1 hour, washed with TTBS (D-PBS, 0.1% Tween 20, 8221840500 Merk Millipore, USA) four times, for 5 minutes. Incubation was performed for 1 hour with 0.1 μg mL^-1^ streptavidin-peroxidase (S5512, Sigma) followed by washing with the corresponding buffer. Detection was performed with the Immobilon Western chemiluminescence HRP substrate (WBKLS0500 Millipore, USA). As a control for non-specific binding, AAL lectin was incubated in the presence of the competitive sugar 0.1 M fucose (Fuc) [32]

## Results and discussion

### Design of mucin detection and quantification assay

The method developed for mucin detection and quantification was implemented as described in Fig 1. Each step was optimized taking into account factors such as buffer selection, the type of lectin used and its loading concentration and mucin concentration (maximum and minimum) in the association step. Two distinct mucins were evaluated during this study: Bovine Submaxillary Gland Mucin (BSM) and human MUC5B.

**Fig 2:**
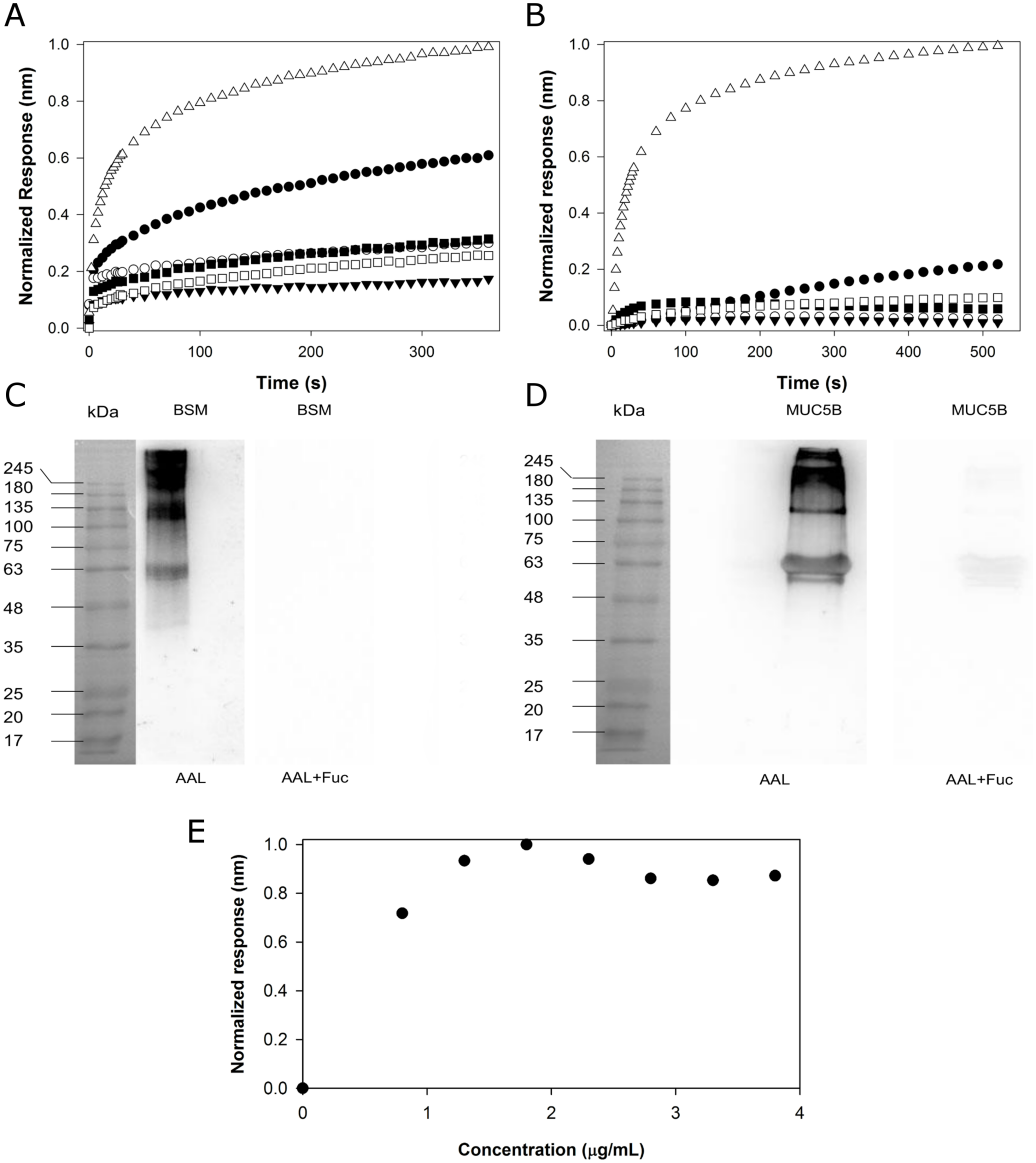
Lectin selection, BSM/MUC5B lectin blotting and loading optimization. Representative curves for association response for BSM **(A)** and MUC5B **(B)** at 1.5 μg/mL for all lectins—MAL (●), PNA (○), SNA (▼), ALL (△), WGA (■), GNA (□) (n = 3). AAL lectin blots containing 2.5 μg of BSM **(C)** and 0.5 μg of MUC5B (**D**). Negative control with competitive sugar, 0.1 M L-Fuc, (AAL + Fuc), is shown for each mucin. **(E)** Optimal loading concentration (1.5 μg mL^-1^) was assessed for AAL lectin ranging 0.8 to 3.8 μg ml^-1^ (n = 6).

A plethora of biotinylated lectins (MAL, AAL, PNA, SNA, WGA, GNA), which bind structures present in their glycans, were evaluated. AAL recognizes fucosylated structures (Fucα1-2Galβ1-4GlcNAcβ-R, Galβ1-4(Fucα1–3)GlcNAcβ-R; R(Fucα1,6)GlcNAc-R), MAL binds α3-linked NeuAc containing structures (NeuAcα2-3Galβ1-4GlcNAcβ1-R), PNA binds the T antigen (Galβ1-3GalNAcα1-Ser/Thr), SNA binds α6-linked NeuAc containing structures (NeuAcα2-6GalNAc/Gal-R), WGA (specific GlcNAc-containing structures, NeuAc) and GNA binds α 1–3 and α 1–6 linked high mannose structures [32]. In order to select the most suitable lectin for mucin detection and quantification, BSM and MUC5B mucins association response to these lectins was evaluated (Fig 2A and 2B). Mucins’ dissociation was also a parameter that was taken into account. However, the dissociation values were negligible when compared to the association.

AAL and MAL lectins presented the highest loading values to the streptavidin biosensor. From these two lectins, AAL was selected for the further experiments due to a higher mucin association response levels since AAL is specific to Fuc-containing structures, present in both mucins in the study [17,20,33]. BSM and MUC5B association response levels for AAL were expected. In order to confirm mucin-lectin binding specificity, lectin blots of BSM and MUC5B with AAL were performed (Fig 2C and 2D) and Fuc, the competitive inhibitor for mucin-AAL binding, was used as negative control [34]. As observed, both mucins were strongly detected with AAL, and the binding was abolished with 0.1 M Fuc, which supported the binding specificity. MUC5B exhibited a higher signal, in comparison with BSM, on the lectin blot even with lower amounts, which could be due to a higher extent of MUC5B fucosylation. Therefore, in order to optimize lectin blot analysis, the amount of MUC5B and BSM loaded was optimized to 0.5 μg and 2.5 μg. This difference may be explained by the different amounts and types of structures containing Fuc, present on both mucins. Additionally, as MUC5B sample presents a higher purity level [8] it can also have an impact on the absolute association response value. This was also observed in octet binding response and will be discussed below.

In order to assess the ideal concentration of immobilized AAL, several concentrations (ranging from 0.8 μg mL^-1^ to 3.8 μg mL^-1^) were loaded onto the biosensor (Fig 2E). For higher concentrations (above 2 μg mL^-1^) the response was not concentration-dependent, presenting an erroneous behaviour, due to biosensor saturation. In the loading step, higher concentrations of the ligand can rapidly saturate the biosensor, whereas the lowest concentrations do not reach saturation. The conditions of an ideal assay should comprise a significant loading signal with a slow initial binding without biosensor saturation. Altogether, the loading concentration selected should be the lowest value, where an acceptable association signal is achieved [27]. Therefore, to avoid biosensor overcrowding and to minimize mucin binding interferences, the optimal lectin concentration selected was 1.5 μg mL^-1^, since it is just before the saturation plateau [27]. To further confirm that AAL-Mucin binding observed in the association responses, binding of lectins to naked-biosensors was also evaluated (S1 Fig). Non-specific bindings to the streptavidin biosensor were observed but with an insignificant intensity when compared to the values for biosensors loaded with AAL lectin.

### Mucin detection and quantification

**Fig 3:**
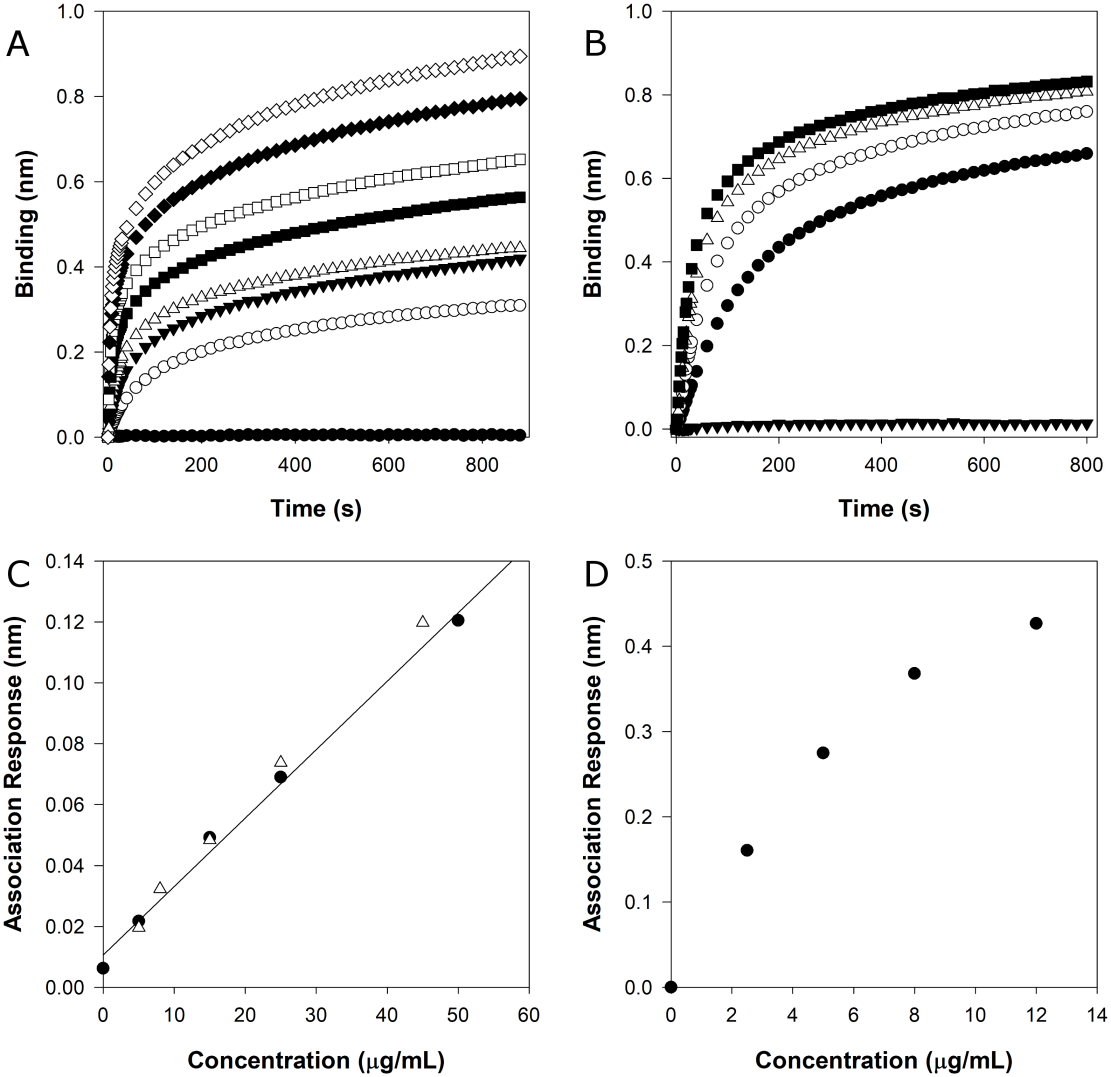
Calibrations curves for BSM and MUC5B and assay validation. Illustrative binding curves for BSM (n = 3) **(A)** and MUC5B (n = 1) **(B)**. BSM calibration curve was performed at a higher concentration curve ranging 25 to 800 μg mL^-1^–800 μg mL^-1^ (◇), 600 μg mL^-1^ (◆), 400 μg mL^-1^ (□), 200 μg mL^-1^ (■), 100 μg mL^-1^ (△), 50 μg mL^-1^ (▼), 25 μg mL^-1^ (○) and 0 μg mL^-1^ (●). MUC5B calibration curve concentration ranged from 2.5 to 12 μg mL^-1^–12 μg mL^-1^ (■), 8 μg mL^-1^ (△), 5 μg mL^-1^ (○), 2.5 μg mL^-1^ (●) and 0 μg mL^-1^ (▼). **(C)** Calibration curve of BSM—full dots (●) represent the values of the calibration curve and the empty triangles (△) correspond to samples with an unknown compared with the calibration curve. **(D)** Calibration curve of MUC5B –full dots (●) represent the values of the calibration curve. The standard error of the estimation associated with the linear regression is 0.07 nm (which corresponds to 2.71 μg mL^-1^) for BSM and 0.054 nm (which corresponds to 1.54 μg mL^-1^) for MUC5B.

To evaluate Biolayer interferometry (BLI) detection and quantification method, association responses of BSM (Fig 3A) and MUC5B (Fig 3B) at different concentrations were analysed. As a first approach, BSM calibration curve was performed using mucin concentration values ranging from 25 to 800 μg mL^-1^ (Fig 3A). However, the response was not linear above 50 μg mL^-1^, probably due to biosensor saturation and mucin-mucin complex binding. Therefore, the calibration curves for BSM studies were performed only in the linear range (until 50 μg mL^-1^) observed in Fig 3C. The dissociation step was also evaluated and for these concentrations the values observed were negligible. The same approach was applied for MUC5B, but using lower concentrations on the standard curve, ranging from 2.5 until 12 μg mL^-1^ (Fig 3B). For this mucin, a nonlinear behaviour was observed above 5 μg mL^-1^(Fig 3D).

In order to evaluate the quantification method, in particular the calibration curve, BSM samples with known concentrations were run against BSM calibration curve (Fig 3C). The values calculated using BLI assay were well correlated with the known values, presenting only slight deviations from the real concentration, which fall in the standard error of the estimation associated with the linear regression.

**Fig 4:**
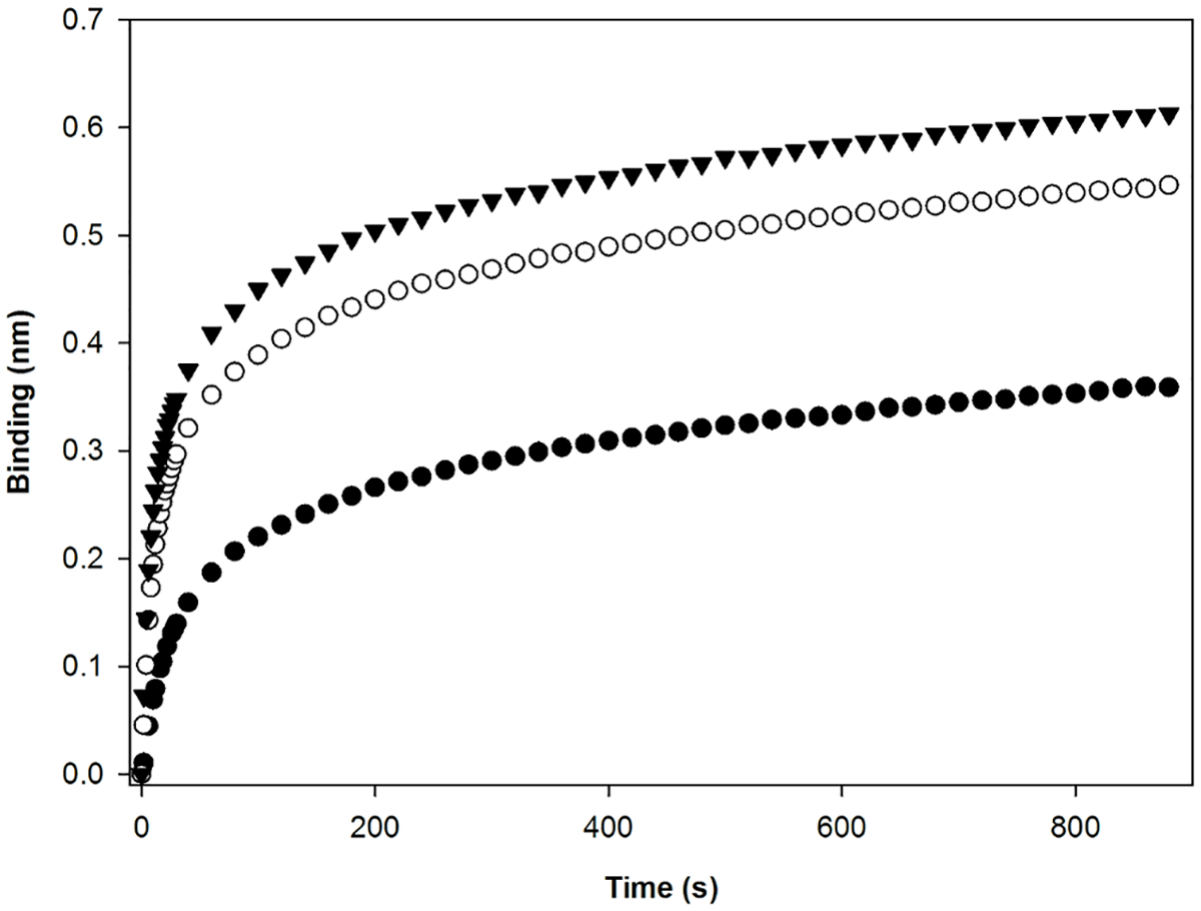
Response comparison of BSM crude sample and after purification. Association of BSM sample, as commercial available (●)and purified using size exclusion chromatography (SEC). Association responses for the two SEC peaks were evaluated, the full inverted triangles (▼) and the empty circles (○) correspond to peaks 1 and 2, respectively.

As discussed previously, MUC5B presented a higher binding response than BSM, probably due to their differences in the purification level. Several studies reported the presence of impurities, or non-mucin biomolecules (e.g. albumin, immunoglobulins and salts), on most commercial mucins, including BSM [35,36]. The major impurity of BSM is bovine serum albumin (BSA) that represents up to 9% of the total mass of commercially available BSM [37]. For that reason, there are numerous purification methods described in the literature that can remove most of these impurities [38]. To understand if the sample purity influences the association response values, BSM sample was purified using size exclusion chromatography. The association response was compared with a non-purified sample and with a purified one. After the purification step, the association response was higher than the one obtained for the commercially available sample (Fig 4). This result confirms that sample purity influences association responses, which was already reported [12]. Therefore, it is important to use appropriate standards for the calibration curves, with a similar purity level and a similar up and downstream processes. This factor is not an issue when we are only detecting the presence of mucins in a fraction, which is of extreme importance in bioprocess development and optimization.

Currently, there are no absolute quantification methods available for mucins, mostly due to their saccharides heterogeneity [5]. For this reason, crossing several analytical methods with different principles is the most suitable approach. Moreover, the detection methods available are time-consuming, an important parameter to take into account during bioprocess development [10]. The method developed here successfully detects different mucins in a wide range of concentrations and at different purification levels. BLI assay is a fast, high throughput and simple method for mucins quantification. Limit of Detection (LOD) and Limit of Quantitation (LOQ) were calculated [28] and compared with the available methods [6][39][40]. For BSM, the LOD and LOQ calculated were 3.54 ± 0.66 μg mL^-1^ (corresponding to 708 ng) and 10.72 ± 2 μg mL^-1^ (corresponding to 2144 ng), respectively. The coefficient of variation is 0.09 for both LOD and LOQ. For MUC5B, the LOD and LOQ calculated were 0.2 μg mL^-1^ (corresponding to 40 ng) and 0.6 μg mL^-1^ (corresponding to 120 ng), respectively. The BLI assay enable us to detect mucins at the same range as in ELISA assays (nanograms) [29]. In addition, this method allowed real-time monitoring of binding interactions and shorter assay development times.

Thus, the established method can help to identify the optimal DSP conditions with the desired yield, binding specificity, and potency [27].

### Competition inhibition assays

Mucin-lectin binding can be exploited to develop mucins’ purification processes [41]. Besides the importance of selecting a lectin with high mucin binding affinity, it is crucial to settle a purification process.

**Fig 5:**
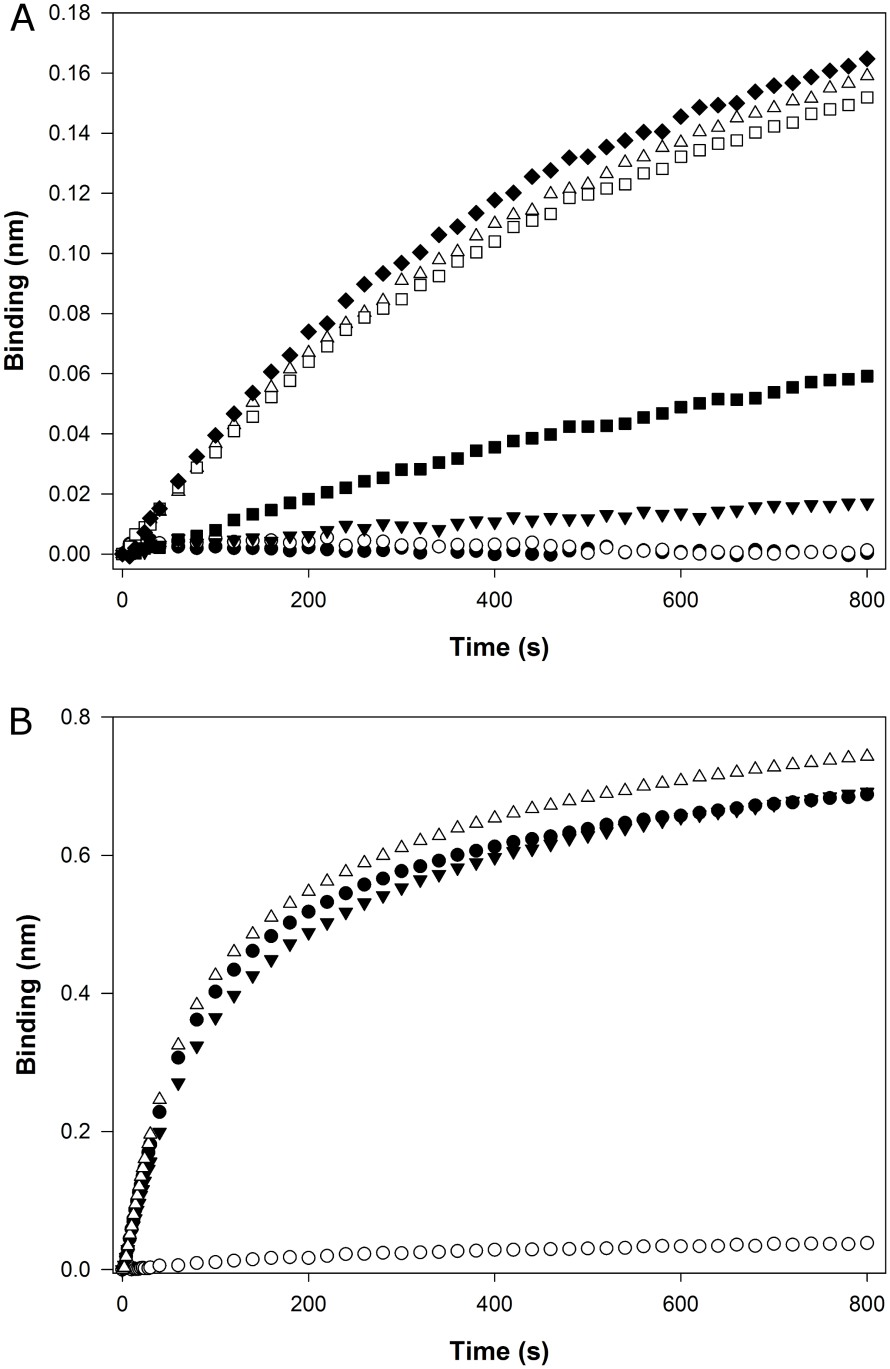
Competition inhibition assays. Competition assay developed for BSM **(A)** (n = 5) and MUC5B **(B)** (n = 2). 0.1 M L-Fuc was used to block the biosensor for both mucins. **(A)** BSM samples were incubated using L-Fuc concentrations ranging from 0.1 M until 1 μM– 0.1 M (●), 10 mM (○), 1 mM (▼), 100 μM (■), 10 μM (□), 1 μM (△) and 0 μM (◆) **(B)** MUC5B samples were incubated using L-Fuc concentrations ranging from 100 μM to 1 μM– 100 μM (○), 10 μM (▼), 1 μM (●) and 0 μM (△).

In order to establish a protocol for mucin purification, using affinity chromatography, the parameters for a possible mucin elution condition were evaluated. Fuc was chosen for this assay since this sugar competes with mucins for AAL binding sites. As a first approach, 0.1 M L-Fuc, which efficiently prevents AAL binding on lectin blotting was used to block AAL binding sites, already loaded in the biosensors, during the second baseline step. However, only this incubation was not sufficient to completely impair mucin binding to AAL. To accomplish a proper blockage, an additional incubation step was performed for BSM and MUC5B samples, using L-Fuc at different concentrations (Fig 5A and 5B).

For BSM mucin, incubation with different L-Fuc concentrations, ranging from 0.1 M until 1 μM was performed (Fig 5A). The minimum Fuc concentration required for partial association inhibition was 100 μM for the sample incubation, and 0.1M for the biosensor. At this concentration there was association, but it was approximately five times lower than the one observed for BSM without Fuc incubation. Fuc concentrations above 100 μM completely inhibited BSM—AAL binding. At 10 and 1 μM, the association was similar to the control sample. The required Fuc concentration values for inhibition were similar to the expected, considering the existing reports for column elution [42,43]. However, some differences can be found depending on the glycoproteins in study. Having established Fuc minimum value for partial inhibition, the incubation of MUC5B with Fuc concentrations ranged from 100 μM to 1 μM were evaluated (Fig 5B). Contrarily to BSM, for MUC5B incubation with 100 μM of Fuc, for the mucin sample and 0.1 M for the biosensor, was enough for total association inhibition. It was observed that the binding response of BSM was approximately ten times lower than MUC5B. However, the affinity of BSM to AAL appeared to be higher as it is necessary higher concentrations of competitor sugar to impair their association. Overall, it was possible to identify the minimal L-Fuc concentration to inhibit AAL-mucin association, for both mucins. The obtained results can be further used in the development of downstream processes for mucins purification, having been established the minimal concentration of L-Fuc used for setting up elution conditions, for example, in an affinity chromatography.

## Conclusion

The increasing interest in mucins is pushing the need for bioprocess development and new characterization tools that are fast and high throughput, robust and easy to set up and that can be used for in-process samples. The methods available for mucins’ detection and quantification do not cope with these requirements: they are not suitable for process development because the assays are time-consuming and it is necessary to cross different tools with different principles to achieve an absolute quantification [11].

The methodology described here uses BLI technology allowing a real-time monitoring and quantification of mucins in a fast, simple and label-free manner. In fact, we were able to decrease the required time for mucins’ detection and quantification in about 5 h, as we moved from an ELISA that takes approximately 5.5 h to a 40 minutes assay. The results indicated that AAL is the most suitable lectin to bind the two model mucins: BSM and MUC5B. Moreover, we were able to detect and quantify samples from distinct purification steps and with different concentrations. The LOD and LOQ obtained, together with the time required to perform the assay showed that the method presents clear advantages compared with the traditional ones. Additionally, competition assay allowed the identification of the minimal Fucose concentration to inhibit AAL-mucin association. These results can be applied for mucin purification strategies in downstream process development, in particular for setting up elution conditions in affinity chromatography. Overall, this tool can be useful in the identification of optimal downstream processing conditions with the desired yield, binding specificity and potency [27], decreasing sample preparation requirements, enhancing throughput with low cost of operation.

## Supporting information

S1 Fig Evaluation of non-specific binding for BSM. Representative association curves for BSM. The full triangles (▲) represent the association response into the streptavidin biosensor loaded with AAL lectin and the empty triangles (△) represent the association response into the naked streptavidin biosensor.
(TIF)
