# Supplementary figures and images for: A detection and quantification label-free tool to speed up downstream processing of model mucins
Source: PLoS One. 2018 Jan 9;13(1):e0190974. doi: 10.1371/journal.pone.0190974 (PMC5760085; doi:10.1371/journal.pone.0190974)

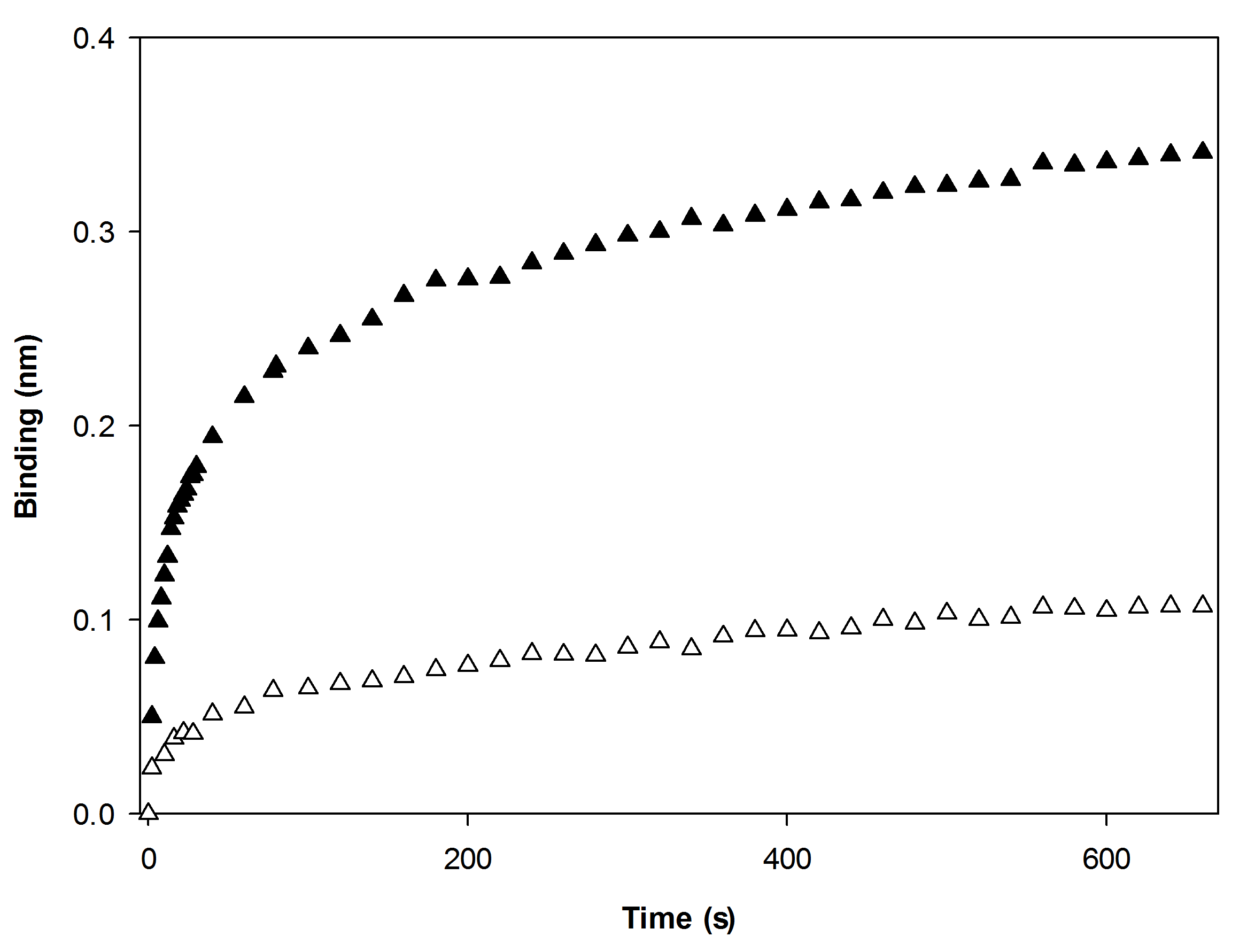

Supplement: S1 Fig — Representative association curves for BSM. The full triangles (▲) represent the association response into the streptavidin biosensor loaded with AAL lectin and the empty triangles (△) represent the association response into the naked streptavidin biosensor. (TIF) [file pone.0190974.s001.tif]
